# Screen Fast, Screen Faster: A Pilot Study to Screen for Depressive Symptoms Using the Beck Depression Inventory Fast Screen in Parkinson's Disease With Mild Cognitive Impairment

**DOI:** 10.3389/fneur.2021.640137

**Published:** 2021-03-08

**Authors:** Saskia Elben, Karina Dimenshteyn, Carlos Trenado, Ann-Kristin Folkerts, Anja Ophey, Patricia Sulzer, Sara Becker, Nele Schmidt, Inken Tödt, Karsten Witt, Inga Liepelt-Scarfone, Rezzak Yilmaz, Elke Kalbe, Lars Wojtecki

**Affiliations:** ^1^Department of Neurology, Center for Movement Disorders and Neuromodulation, Medical Faculty, Heinrich Heine University, Düsseldorf, Germany; ^2^Institute of Clinical Neuroscience and Medical Psychology, Medical Faculty, Heinrich-Heine-University, Düsseldorf, Germany; ^3^Systems Neuroscience and Neurotechnology Unit, Faculty of Medicine, Saarland University and HTW Saarland, Homburg, Germany; ^4^Medical Psychology | Neuropsychology & Gender Studies, Center for Neuropsychological Diagnostics and Intervention (CeNDI), Faculty of Medicine and University Hospital Cologne, University of Cologne, Cologne, Germany; ^5^German Center of Neurodegenerative Diseases, Eberhard Karls University Tuebingen, Tübingen, Germany; ^6^Clinical Neurodegeneration, Hertie-Institute for Clinical Brain Research, Eberhard Karls University Tuebingen, Tübingen, Germany; ^7^Department of Neurology, Christian-Albrechts University of Kiel, Kiel, Germany; ^8^Department of Neurology and Research Center Neurosensory Science, Carl von Ossietzky University Oldenburg, Oldenburg, Germany; ^9^Studienzentrum Stuttgart, IB Hochschule, Stuttgart, Germany; ^10^Department of Neurology, University of Ankara Medical School, Ankara, Turkey; ^11^Department of Neurology and Neurorehabilitation, Hospital zum Heiligen Geist, Kempen, Germany

**Keywords:** Parkinson's disease, depression, mild cognitive impairment, BDI-FS, BDI-II

## Abstract

**Objective:** Depressive symptoms have a high prevalence in patients with Parkinson's disease (PD) and are associated with cognitive dysfunction. Especially in PD with mild cognitive impairment (MCI), a time-efficient and valid instrument for the assessment of depression primarily focusing on psychological symptoms and disregarding confounding somatic symptoms is needed. We performed an examination of the psychometric properties of the Beck Depression Inventory II (BDI-II) and the Beck Depression Inventory Fast Screen (BDI-FS).

**Methods:** The sample consisted of 64 patients [22 females and 42 males, mean age: 67.27 years (*SD* = 7.32)]. Depressive symptoms were measured in a cohort of PD patients with MCI. For the BDI-II and BDI-FS the psychometric concepts of internal consistency, convergent validity and diagnostic agreement were assessed.

**Results:** Patients gave higher ratings on test items addressing somatic symptoms than those addressing non-somatic ones. The correlation between the absolute total scores of the BDI-II and the BDI-FS was significant (*r* = 0.91, *p* < 0.001), which indicated convergent validity. The Cronbach's alpha values indicated adequate internal consistencies for both measures (BDI-II: 0.84; BDI-FS: 0.78). There was a higher than chance level agreement of diagnoses of the two questionnaires, measured by Cohen's kappa (0.58, *p* < 0.001). The agreements between previous diagnosis of depression and the diagnoses of the BDI-II/BDI-FS were also significantly higher than chance level (BDI-II: 0.34, *p* = 0.007, BDI-FS: 0.39, *p* = 0.002). Additional AUC analysis across different cutoffs showed that performance of BDI-FS was better than BDI-II, supporting the observation of an equivalent or better performance of BDI-FS than BDI-II. Importantly, AUC analysis confirmed that a cutoff = 4 for BDI-FS was suitable in the considered sample of patients with PD-MCI.

**Discussion:** In a cohort of PD-MCI, the BDI-FS demonstrates adequate psychometric properties in comparison to the BDI-II and can be used as a screening measure for assessing depression in cognitively impaired PD patients, focusing solely on psychological symptoms. Still, further research is needed to validate this instrument.

## Introduction

Parkinson's disease (PD) is primarily known to bear motor symptoms such as tremor, bradykinesia, rigidity, and postural instability (1). However, not only motor symptoms play a role in PD, there is also a high prevalence of non-motor symptoms such as depression (2). A depressive condition in PD has substantial clinical relevance and a high impact on patients' quality of life (3). Thus, efficient and accurate instruments for the diagnosis of depressive symptoms in PD for clinical practice are of utmost importance. Furthermore, as previously described by other studies, an association exists between depression and cognitive dysfunction. Mild cognitive impairment (MCI) and dementia occur frequently in PD patients, and the presence of MCI increases the risk of developing a fully pronounced dementia (1, 4). The Global Parkinson's Disease Survey demonstrated that depression is the most important predictor for cognitive dysfunction in PD (5). This finding emphasizes the relevance of efficient detection of depression in PD with MCI, especially for avoiding further decompensation of the cognitive impairment.

A commonly implemented test for the assessment of depression is the Beck Depression Inventory, which is currently available in a revised second version (BDI-II) (6). The test was shown to have adequate diagnostic properties in PD samples (7–9). Nevertheless, there are several aspects about the BDI-II that may be detrimental for screening, especially in outpatient or primary care settings. First, it is extensive and its length can be shortened for the sake of time efficiency and less cognitive demand. Second, and most importantly in patient populations such as PD, the use of items addressing somatic symptoms can be confounded by symptoms that are caused purely by PD and not by a depressive condition, thus resulting in false positive diagnoses (10, 11). When scrutinized in detail, somatic items from the BDI-II such as for example *Loss of Energy, Changes in Sleeping Pattern, Changes in Appetite*, or *Tiredness or Fatigue* can represent a PD symptom and not depict an underlying depression.

As an alternative measure with regard to the aforementioned issues, Beck et al. proposed the Beck Depression Scale Fast Screen (BDI-FS) (12). It contains seven items from the BDI-II that exclusively address psychological symptoms and was designed specifically for implementation in patients with somatic medical conditions. Previous research demonstrated adequate psychometric properties in various diseases, such as multiple sclerosis, end-stage renal disease, pain syndrome, and stroke (13–16). The psychometric properties in PD samples have not yet been investigated.

The aim of the present study was to assess the psychometric properties of the BDI-FS in a sample of PD patients with MCI. The following aspects were investigated:

- Internal consistency of the BDI-FS as a measure of reliability
- Convergent validity by measuring the correlation between the BDI-FS and the BDI-II
- Diagnostic validity by comparing scoring above a defined cutoff indicating a clinical condition for major depression via BDI-FS and BDI-II
- Diagnostic validity by comparing the scoring above a defined cutoff indicating a clinical condition for major depression via BDI-FS diagnosis and current clinical diagnosis of depression according to the ICD-10 (17).

## Methods

### Sample

The data used here was taken from the multicentric study “Training Parkinson Patients' Cognition” (TrainParC, German Registry for Clinical Studies no. 00010186), involving the university hospitals of Düsseldorf, Cologne, Kiel, and Tübingen. The conduction was approved by the ethics committee of all involved universities.

Sixty-four patients were included into the study. Main inclusion and exclusion criteria as well as the MDS specific guidelines for PD-MCI level I and level II categories (18) are listed in Table 1.

Table 1Inclusion and exclusion criteria, MDS specific guidelines for PD-MCI level I and level II categories and neuropsychological test results of the study sample.

**Table:** 

**Inclusion criteria**	**Exclusion criteria**
(i) Age between 50 and 85 years	(i) Clinical PDD diagnosis or outcomes in the Pill-Questionnaire indicating caregiver dependency
(ii) PD diagnosis according to the UK Brain Bank Criteria	(ii) Depression (operationalized with Beck Depression Inventory II ≥20 points)
(iii) Self-reported cognitive impairment assessed with the Subjective Cognitive Impairment questionnaire and/or objective cognitive impairment assessed with the Montreal Cognitive Assessment <26 points	(iii) Acute suicide tendency
(iv) PD-MCI according to Movement Disorders Society (MDS) task force Level-II criteria	(iv) Severe comorbidities affecting life expectancy, medication, or quality of life
(v) At least 1 year since PD diagnosis	(v) Severe fatigue
(vi) Stable medication within 4 weeks before screening	(vi) Prominent impulse control disorder or dopamine dysregulation syndrome
(vii) Written informed consent	(vii) Acute psychosis or psychotic episode in the last 6 months before study participation
	(viii) Dementia medication
	(ix) Participation in other treatment studies within the last 2 months before study participation
	(x) Deep brain stimulation
	(xi) Pregnancy or nursing period

**Table:** 

**MDS specific guidelines for PD-MCI level I and level II categories**	**Neuropsychological test results of the study sample**		
A. Level I (abbreviated assessment)	**Attention**	***M***	***SD***
•Impairment on a scale of global cognitive abilities validated for use in PD or	d2-R concentration performance	−1.55	1.01
	d2-R errors	−0.37	1.335
•Impairment on at least two tests, when a limited battery of neuropsychological tests is performed (i.e., the battery includes less than two tests within each of the five cognitive domains, or less than five cognitive domains are assessed)	**Working memory**		
	Letter-number sequencing	0.095	0.895
	Digit span backwards	−0.23	1.11
B. Level II (comprehensive assessment) •Neuropsychological testing that includes two tests within each of the five cognitive domains (i.e., attention and working memory, executive, language, memory, and visuospatial) •Impairment on at least two neuropsychological tests, represented by either two impaired tests in one cognitive domain or one impaired test in two different cognitive domains •Impairment on neuropsychological tests may be demonstrated by: Performance ~ 1–2 SDs below appropriate norms or Significant decline demonstrated on serial cognitive testing or Significant decline from estimated premorbid levels	**Executive functions**		
	Semantic word fluency	0.415	1.16
	Phonemic word fluency	0.305	1.01
	MCST categories	−0.865	1.015
	Key search—raw score	11.275	3.295
	**Language**		
	Boston nming test	−0.09	1.31
	ACL speech comprehension—raw score	17.635	1.155
	**Memory**		
	CVLT learning performance	−1.19	1.25
	CVLT long delay free recall	−1.215	1.105
	ROCFT delayed free recall	0.19	1.06
	**Visuocognition/visuospatial**		
	ROCFT figure copy	0.24	1.44
	Bnton judgement of line orientation	−0.615	1.405

*Data of the baseline neuropsychological test results presented as mean standardized z-scores or raw scores and standard deviations, with higher values indicating better performance. MCST, Modified Wisconsin Card Sorting Test; ACL, Aphasia Check List; CVLT, California Verbal Learning Test; ROCFT, Rey-Osterrieth Complex Figure Test*.

Furthermore, patients were also classified according to the stages of PD by Hoehn and Yahr (19). For detailed information regarding the main study please refer to Kalbe et al. (20).

### Tests and Further Assessments

#### BDI-II

The German version of the BDI-II (6, 21) was used to assess depressive symptoms. It consists of 21 items, each of which is rated on a 4-point Likert scale. The point value of a single item ranges from zero to three points. The items represent the symptoms of a depressive episode defined by the Diagnostic and Statistical Manual of Mental Disorders, Fourth Edition (DSM-IV) (22). Higher scores indicate more severe symptoms, scores of 14 and above indicate at least a mild condition, and a maximum of 63 points can be achieved (6).

#### BDI-FS

The BDI-FS (12, 23) is a shortened version of the BDI-II consisting of 7 items, including *Sadness, Pessimism, Past Failure, Loss of Pleasure, Self-Dislike, Self-Criticalness*, and *Suicidal Ideation*. The item format is identical to that of the long version. Again, higher scores indicate a more severe condition with four being the cutoff for clinical significance. The maximum score obtained on the BDI-FS is 21 points. The BDI-FS sum score was obtained by adding the item scores of the respective BDI-II items.

#### Medical History of Depression

Participants were questioned concerning previous medical diagnosis of depression and whether it was still present or in remission. Medical professionals in Germany are obliged to pose diagnoses based on the criteria described in the International Classification System of Diseases, Version 10 (ICD-10) (24).

### Statistical Analysis

The total scores of the BDI-II and the BDI-FS were correlated by use of Spearman correlations. Cronbach's alpha index was calculated to assesses the internal consistency of the two measures. The diagnoses were compared by implementing a Cohen's kappa, which indicates the diagnostic agreement between the two tests. Then, the BDI-II and the BDI-FS and clinical diagnoses were compared to the presence of a diagnosis of depression in the patients' anamnesis. *P*-values below 0.05 were considered significant. All calculations were performed using IBM SPSS Statistics, Version 22.0 (25).

### AUC Analysis

In order to gain further insight into sensitivity and specificity characteristics as well as performance of BDI-FS and BDI-II across a range of cutoffs, receiving operator curves (ROC) on the basis of a logistic regression model (dependent variable: clinical diagnosis of depression; independent variable: BDI-FS or BDI-II score classification for a given cutoff) and “area under ROC” (AUC) were calculated by using MATLAB 8.5, The Mathworks, Inc., Massachusetts, US. Note that AUC quantifies how much a test score for a given cutoff is able to distinguish between depression and non-depression state in a subject. AUC ranges in value from 0 and 1. A test score whose predictions are 100% correct has an AUC of 1.0, whereas a value of 0.5 for AUC indicates that the test score has no discriminatory ability.

## Results

The sample consisted of 64 patients [22 female and 42 male, mean age: 67.27 years (*SD* = 7.32)], with an average of 13.70 years of education (*SD* = 3.37). The mean age at PD onset was 58.33 (*SD* = 11.40). Eight patients were in Hoehn & Yahr stage 1, 40 in stage 2, 15 in stage 3 and one in stage 4. The descriptive statistics for the scores of both questionnaires are presented in Table 2.

**Table 2:** Descriptive statistics of the BDI-II and BDI-FS.

**Statistics**	**BDI-II**	**BDI-FS**
Kolmogorov-Smirnov test	0.170	>0.000
Mean	8.84	2.06
Standard deviation	5.03	2.12
Median	8	2
Range (minimum and maximum value)	0–19	0–8

The correlation between the total scores of the BDI-II and the BDI-FS was 0.87 (*p* < 0.001). Cronbach's alpha for the BDI-II was 0.73 and 0.71 for the BDI-FS. The agreement of diagnoses of the two questionnaires, measured by Cohen's kappa, was significant with a value of 0.59 (87%, *p* < 0.001). The agreement between a current diagnosis of depression in the medical history and the diagnosis of the BDI-II was significantly higher than chance with a Cohen's kappa of 0.34 (81%, *p* = 0.007). The BDI-FS also agreed significantly more often than chance with a present diagnosis of depression, as indicated by a significant Cohen's kappa of 0.39 (81%, *p* < 0.002).

**Figure 1:**
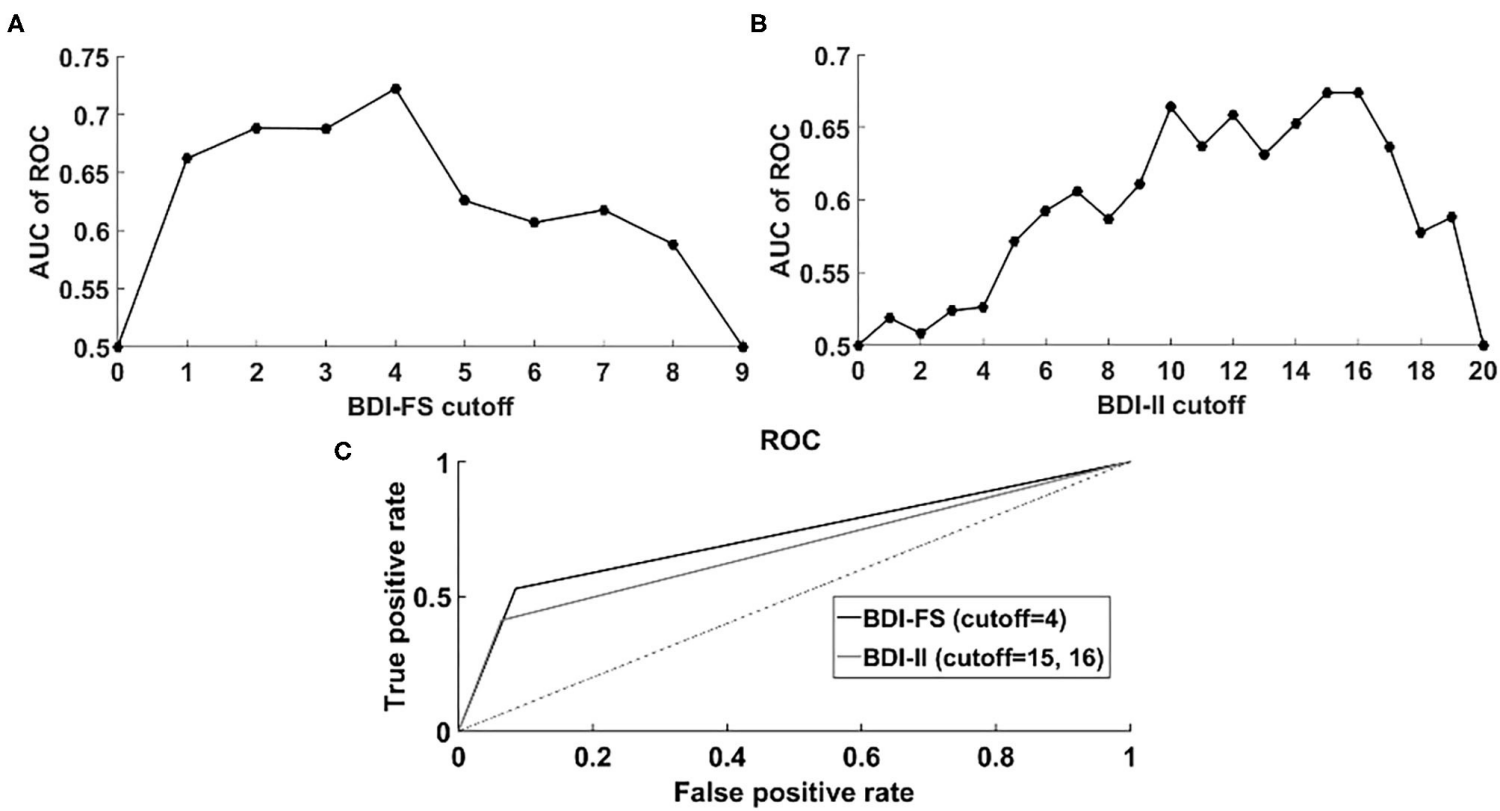
“Area under ROC” (AUC) analysis. **(A)** AUC of ROC corresponding to BDI-FS across different cutoffs: 0–9. It is noticeable that highest performance is achieved at cutoff = 4; **(B)** AUC of ROC corresponding to BDI-FS across different cutoffs: 0–20. It is noticeable that highest performance is achieved at cutoff = 15 and 16; **(C)** ROC of BDI-FS (cutoff = 4) corresponding to logistic regression model: depression-state = 2.4927*BDI-FS −1.6818, *p* < 0.001 (coefficient and intercept) and ROC of BDI-II (cutoff = 15 and 16) corresponding to logistic regression model: depression-state = 2.3289*BDI-II −1.4816, *p* = 0.0026 (coefficient), *p* < 0.001 (intercept). For the considered sample of PD-MCI patients, better performance of BDI-FS than BDI-II was revealed.

AUC analysis revealed maximum performance of BDI-FS (AUC = 0.7222) at cutoff = 4 (Figure 1A), while for BDI-II such performance (AUC = 0.6740) occurred at cutoff = 15 and 16 (Figure 1B). Thus, for the addressed sample of PD-MCI patients, BDI-FS showed better performance in distinguishing a depression state than BDI-II at the mentioned cutoffs (Figure 1C). Both BDI-FS and BDI-II showed better performance than just a random selection (AUC = 0.5). In particular, ROC of BDI-FS at cutoff = 4 revealed low “sensitivity” [true positive rate (TPR)] and high “specificity” [1-false positive rate (FPR)] at threshold = 0.6923 as well as high sensitivity and low specificity at threshold = 0.1569. ROC of BDI-II at cutoff = 15 and 16 revealed low sensitivity and high specificity at threshold = 0.7 as well as high sensitivity and low specificity at threshold = 0.1852.

## Discussion

The present study aimed to define the psychometric properties of the BDI-FS in a PD-MCI population. Our results indicate adequate psychometric properties. The BDI-FS strongly correlates with the BDI-II. It shows an adequate internal consistency, while the BDI-II has a slightly better value (26). The agreement between both measures is significantly higher than chance with a value that can be classified as moderate (26). The agreements of the BDI-II and the BDI-FS with a present diagnosis of depression as indicated by the patient's history can both be classified as fair according to the common norm—the value of the BDI-FS even being slightly larger (27).

One of the most obvious positive characteristics of the BDI-FS is its brevity. Neitzer et al. found that those patients who did not complete the whole BDI-II assessment were significantly older (15). Regarding the rather high mean age of PD patients and potential deficits in language processing, the screening for depression should be kept at a minimum length (26, 28). With a short questionnaire, the resulting validity would not be compromised due to neuropsychological or motivational aspects. As indicated by the high correlation between the two measures, the BDI-FS is an acceptable alternative if time or cognitive resources are a relevant issue.

AUC analysis across different cutoffs showed that performance of BDI-FS was better than BDI-II, thus supporting the observation of an equivalent or better performance of BDI-FS than BDI-II for the considered sample of PD-MCI patients. The fact that BDI-II presented highest performance at cutoffs 15 and 16 is consistent with previous criteria (cutoff >14) as reported in the case of mild condition of depression (6). Importantly, AUC analysis confirmed that a cutoff = 4 for BDI-FS was suitable in the considered sample of patients with PD-MCI.

**Figure 2:**
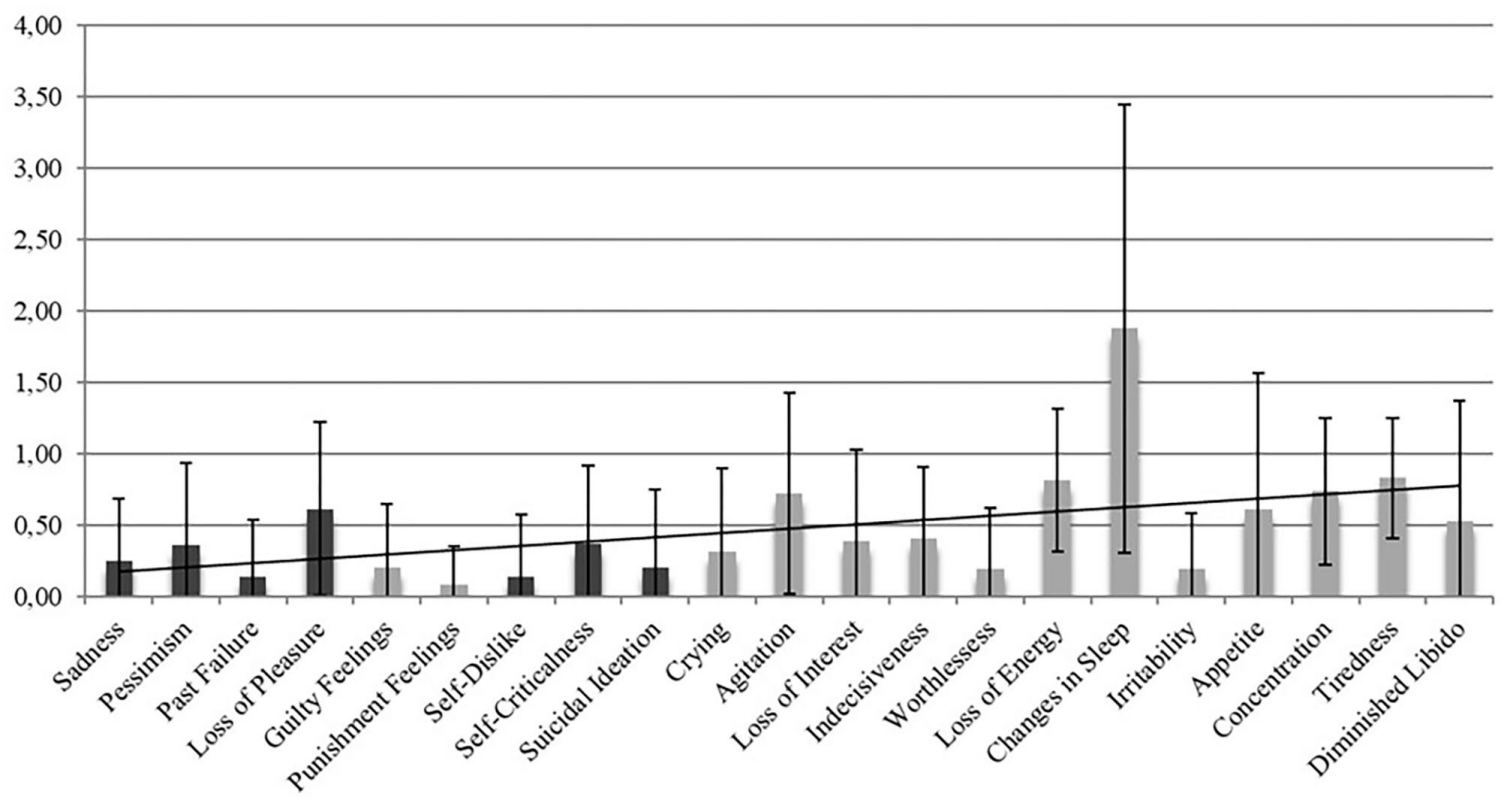
Mean item score values for all items of the BDI-II. The x-axis represents the 21 items of the BDI-II and the 7 BDI-FS items *Sadness, Pessimism, Past Failure, Loss of Pleasure, Self-Dislike, Self-Criticalness, and Suicidal Ideation (dark columns)*. The y-axis depicts the mean scores (and standard deviation) on the individual items with scores ranging from 0 to 3. The items in the second half of the questionnaire address somatic symptoms and mostly received higher scores in our sample than the non-somatic items of the first half.

The BDI-FS deliberately omits the use of somatic items to avoid confounding effects by overlapping symptoms that may stem from a somatic disease rather than depression itself (12). Regarding the spectrum of possible non-motor symptoms in PD, there is a multitude of symptoms that can also count as a somatic symptom of depression, e.g., insomnia, gastrointestinal problems, sexual dysfunction or fatigue (10, 29). In our sample, the items with the highest mean scores were *Changes in Sleeping, Concentration Difficulties, Tiredness or Fatigue*, and *Loss of Energy*. Overall, as Figure 2 shows, the items addressing somatic symptoms, which can be found in the second half of the questionnaire, mostly scored higher than those addressing non-somatic items. This suggests that somatic symptoms can lead to false positive results when the full version of the BDI-II is implemented.

For the purpose of this study, we calculated the BDI-FS score by adding the item scores of the respective BDI-II items. Thus, we cannot guarantee the same results if the patients would have received the BDI-FS as a separate questionnaire. Effects regarding item order and questionnaire length might be a relevant factor. Future studies should take this into account and consider applying the BDI-FS separately.

One potential limitation of the present study is the selectivity of the sample. Whether the results can be transferred to a general PD population, which also includes cognitively unimpaired or demented patients, remains to be scrutinized by future studies. Further research is required to clarify the psychometric properties of the BDI-FS in other, possibly broader PD samples. Furthermore, a moderate or more severe degree of depression was one of the exclusion criteria for the main study in order to prevent interactions between cognitive performance and psychopathology. Therefore, our results can only be transferred to milder forms of depressive states. The psychometric properties in more severe depressed patients are yet to be reviewed.

Furthermore, patients with PD may present somatic symptoms that are not caused by depression, but they may present prototypical somatic symptoms of depression. Not exploring these symptoms could cover up depressive symptoms present in patients, possibly leading to false negative results. Additional analysis of positive and negative predictive value should be addressed by further research.

Even with regard to the overall adequate psychometric properties of the BDI-FS and the BDI-II in PD, it is important to emphasize that their use is appropriate for screening purposes only. If a secure diagnosis is required, neither of the two questionnaires can replace a complete diagnostic procedure. The diagnostic criteria should always be confirmed by a trained clinician in a (semi) structured interview to ensure no confounding effect of possible cognitive or somatic biases, to which depressed patients are particularly prone (30). In the present study, the diagnoses of depression that were used for calculation of the diagnostic validity of the BDI-FS were derived from the patients' medical histories and reports, but no clinical (semi) structured interview was performed. Furthermore, we compared the diagnosis resulting from the assessment by BDI-II or BDI-FS with previous diagnosis of depression in the medical history. As mentioned above, in Germany such a diagnosis is usually based on the criteria posed by the ICD-10 rather than the DSM-V. The questionnaires used are based on the latter. Although both ICD-10 and DSM-V mostly rely on the same symptoms for the diagnosis of a depressive state, the criteria are not identical and the descriptions differ in wording. These factors could account for the relatively low agreement values with the questionnaires, as we cannot guarantee for the validity of these diagnoses. To ensure maximum validity of the diagnostic criterion, future studies should include an assessment of depressive symptoms based on the DSM-V by a trained clinician as part of the procedure.

Finally, to establish possible advantages or disadvantages of the BDI-FS, it can be compared to other frequently administered depression scales. For instance, one test constructed for use in similar populations as the one examined in the present study is the Geriatric Depression Scale (31). One possible aspect for future studies could lie on comparing its psychometric values to those of the BDI-FS, especially with regard to the different item formats (dichotomous vs. Likert scale).

In summary, the BDI-FS shows satisfactory reliability, as well as validity as demonstrated by the correlation and agreement with the full version of the BDI-II. The agreement rates with a current diagnosis of depression are acceptable, although the diagnosis was not assessed as part of the study. A diagnostic interview should be included in future studies of the BDI-FS in PD, preferably in samples of various cognitive statuses. Although the BDI-FS cannot replace a complete diagnostic procedure, it may be used as a shorter alternative to the BDI-II in contexts where time efficiency, somatic symptomatology, and minimum cognitive load are relevant, such as in primary care or outpatient settings.

## Data Availability Statement

The datasets used and/or analyzed during the current study are available from the corresponding author on reasonable request.

## Ethics Statement

The studies involving human participants were reviewed and approved by Ethics Committee of the Heinrich-Heine University Hospital Düsseldorf, Germany. The patients/participants provided their written informed consent to participate in this study.

## Author's Note

Non-motor functions in Parkinson's disease are currently discussed attentively. In particular, depressive symptoms are a matter of ongoing capturing debate, since they have a high prevalence in patients with Parkinson's disease and can be associated with cognitive dysfunction. Therefore, time-efficient and valid instruments for the assessment of depression in such a population are needed. However, popular depression rating scales often include somatic items, which may result in erroneous depression diagnosis in diseases such as Parkinson's disease, presenting with somatic symptoms due to the core disease and not due to depression. Furthermore, not all available psychometric tests have been validated for use in specific populations. To these ends, we examined the psychometric properties of the Beck Depression Inventory II and the Beck Depression Inventory Fast Screen. Our results revealed that the Beck Depression Inventory Fast Screen demonstrates adequate psychometric properties in comparison to the Beck Depression Inventory II and thus is a valid tool for assessment of depression in parkinsonian patients.

## Author Contributions

SE, KD, LW, and EK: conception or design of the work. SE, KD, LW, KW, EK, A-KF, AO, NS, IT, IL-S, PS, SB, and RY: data collection. KD, CT, SE, LW, IL-S, and PS: data analysis and interpretation. SE, KD, and CT: drafting the article. SE, KD, CT, LW, KW, IT, EK, A-KF, AO, IL-S, PS, SB, and RY: critical revision of the article. SE, KD, CT, LW, EK, A-KF, AO, KW, NS, IT, IL-S, PS, SB, and RY: final approval of the version to be published.

## Conflict of Interest

AK-F has received a grant from the German Parkinson Society and honoraria from ProLog Wissen GmbH, Cologne, Germany, and pro audito Switzerland, Zürich, Switzerland. NS has received grants from the German Federal Ministry of Education and Research. KW has received grants for the German Research Foundation, the German Federal Ministry of Education and Research, and received speaker honoraria from BIAL, BAYER, Medtronic, Boston Scientific, Novartis, Desitin, and the German Society of Neurology. IL-S has received grants from the Parkinson Fonds Deutschland gGmbH, Janssen Pharmaceutical Companies of Johnson & Johnson, European Commission, H2020-TWINN-2015, and the Michael J. Fox Foundation. EK has received grants from the German Ministry of Education and Research, Parkinson Fonds Deutschland gGmbH, and the German Parkinson Society and honoraria from Oticon GmbH, Hamburg, Germany; Lilly Pharma GmbH, Bad Homburg, Germany; Bernafon AG, Bern, Switzerland; and Desitin GmbH, Hamburg, Germany. LW has received honoraria from Meda, Boehringer, Cephalon Pharma, TEVA, Desitin, AbbVie St. Jude Medical / Abbott, and Medtronic and grants from HHU Düsseldorf, DFG Forschergruppe (FOR 1328), ERANET Neuron/BMBF (TYMON 01EW141), German Parkinson's Disease Association (dPV), Parkinson Fonds Germany, and Hilde Ulrichs Stiftung für Parkinsonforschung. The remaining authors declare that the research was conducted in the absence of any commercial or financial relationships that could be construed as a potential conflict of interest.
